# Evaluating the use of a customized digital navigation program to optimize bowel preparation in pediatric colonsocopy

**DOI:** 10.3389/fped.2023.1271222

**Published:** 2023-10-09

**Authors:** Lauren Dankner, Jose Antonio Quiros, Diana Volpert, Ashish Atreja

**Affiliations:** ^1^ Division of Pediatric Gastroenterology, Valley Hospital, Ridgewood, NJ, United States; ^2^ Division of Pediatric Gastroenterology, Icahn School of Medicine at Mount Sinai, New York, NY, United States; ^3^ Chief Information and Digital Health Officer, UC Davis Medical Center, Sacramento, CA, United States

**Keywords:** pediatric endoscopy, endoscopy, digital health, bowel preparation, gastroenterology

## Abstract

**Introduction:**

Adequate bowel preparation is essential for optimal colonoscopy diagnosis and/or intervention. However, suboptimal bowel preparation occurs in as many as 1 in 3 pediatric colonoscopies, leading to missed diagnoses, procedural complications, wasted resources, and increased costs. We aimed to evaluate the effect of an automated Pediatric Colonoscopy Digital Navigation Program (PC-DNP) on the quality of colonoscopy preparation among pediatric patients.

**Methods:**

The PC-DNP sent patients timely weight-based bowel preparation instructions, video and text-based educational modules, logistical information, and appointment reminders prior to their scheduled diagnostic and/or therapeutic colonoscopies. Physician reported bowel preparation quality among patients/caregivers who were prescribed the PC-DNP were compared to bowel preparation quality of a historical sample of patients/caregivers who received standard care instructions.

**Results:**

We found that the PC-DNP group had significantly higher bowel preparation quality than the standard care group.

**Conclusions:**

These results demonstrated that automated DNPs may be easily implemented into the pediatric gastroenterologists' practice and may help streamline and improve bowel preparation in pediatric patients.

## Introduction

Colonoscopy is an endoscopic procedure commonly performed to diagnose and treat pediatric gastrointestinal (GI) conditions including abdominal pain, diarrhea, and hematochezia. Adequate bowel preparation prior to colonoscopy is essential for optimal diagnosis and/or intervention. However, suboptimal bowel preparation occurs in as many as 1 in 3 pediatric colonoscopies, leading to missed diagnoses, procedural complications, and increased costs (1). Ensuring that patients/caregivers fully understand bowel preparation instructions is a key component of achieving adequate bowel preparation (2). Common barriers to good quality bowel preparation include difficulty understanding lengthy instructions, misplacement of the instructions, confusion regarding mixing of the preparation, and lack of clarity on the expected stool appearance (3). Currently, GI practices try to address these barriers through manual interventions and frequent communication by nurses and practice staff, which is labor intensive and provides suboptimal patient experience and outcomes. Digital health technology has recently emerged as an effective approach to addressing such barriers and helping patients better prepare for colonoscopies.

Digital Navigation Programs (DNPs) have been shown to significantly improve colonoscopy preparation quality in adult patients undergoing colonoscopies by providing automated pre-procedure navigation and instructions directly to patients via text-messages, email and/or through patient portal (4–6). These automated messages include time-based bowel preparation instructions, dietary modification instructions, educational content, and appointment reminders. They are tailored to users and often include facility-specific information. Despite the success of DNPs in improving colonoscopy preparation quality in adults, to date, few studies have investigated their effects in the pediatric population.

In this study, we evaluated the impact of a Pediatric Colonoscopy DNP (PC-DNP) on the colonoscopy preparation quality of pediatric patients. The PC-DNP sent patients timely weight-based bowel preparation instructions, video and text-based educational modules, logistical information, and appointment reminders in preparation for their scheduled diagnostic and/or therapeutic colonoscopy. This study was intended to compare the bowel preparation quality of those who utilized the PC-DNP to those who received standard care instructions.

## Methods

### Study design & participants

**Figure 1:**
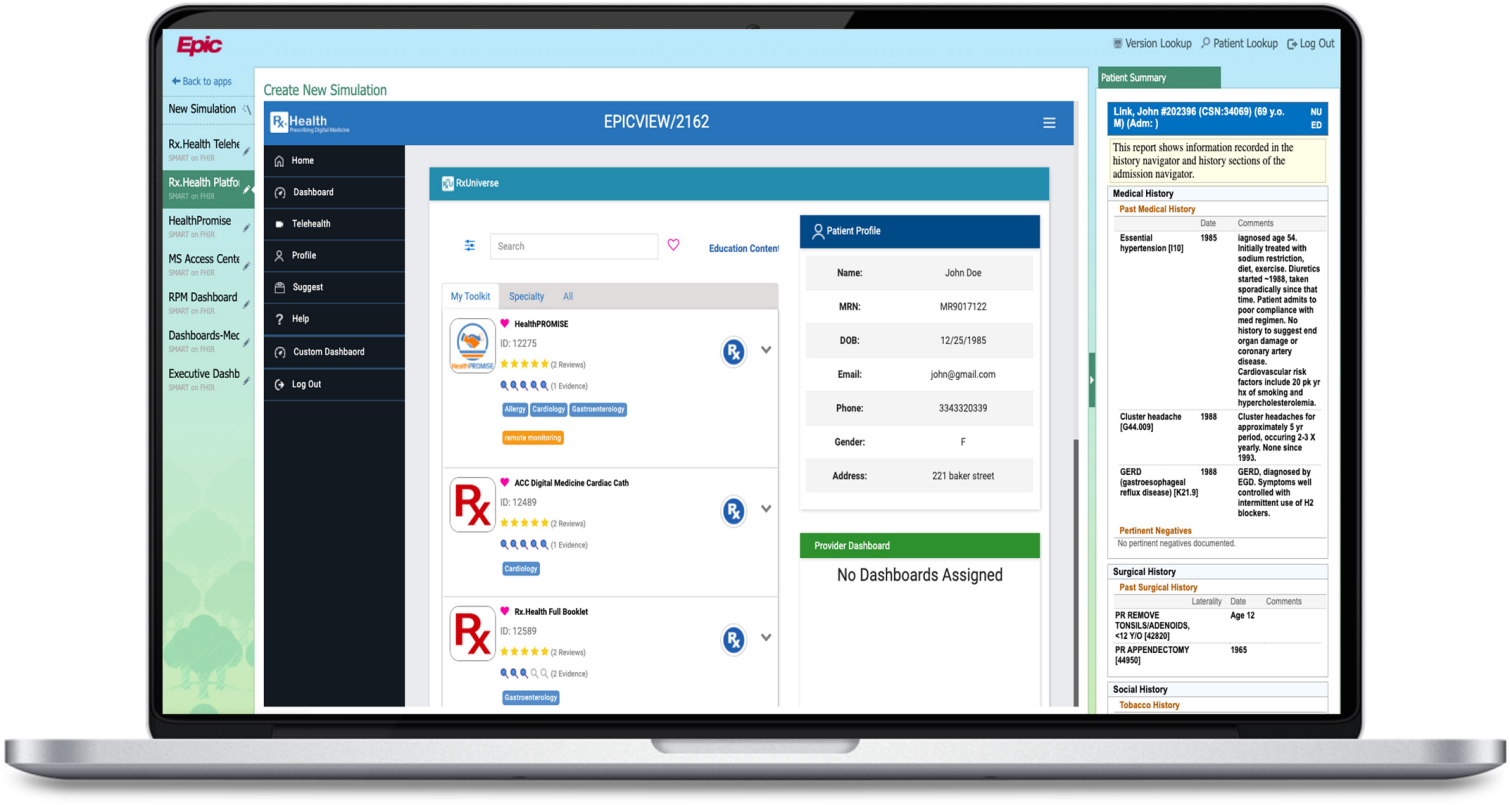
Prescriber view of customizable DNP.

Included in this study were pediatric patients age 20 years and below who underwent diagnostic and/or therapeutic colonoscopies at The Valley Hospital. The Valley Hospital implemented this initiative as part of Digital Transformation Network led by Rx.Health, NY in partnership with American Gastroenterological Association (AGA). Physician-reported bowel preparation quality of patients/caregivers who were prescribed the PC-DNP (from an EHR connected Digital Health Formulary) (Figure 1) were compared retrospectively to bowel preparation quality of a historical sample of patients/caregivers who received standard care cleanout instructions. The latter were weight- based instructions of the same dosing and instructions given as a print out to the parent or guardian prior to the procedure.

**Figure 2:**
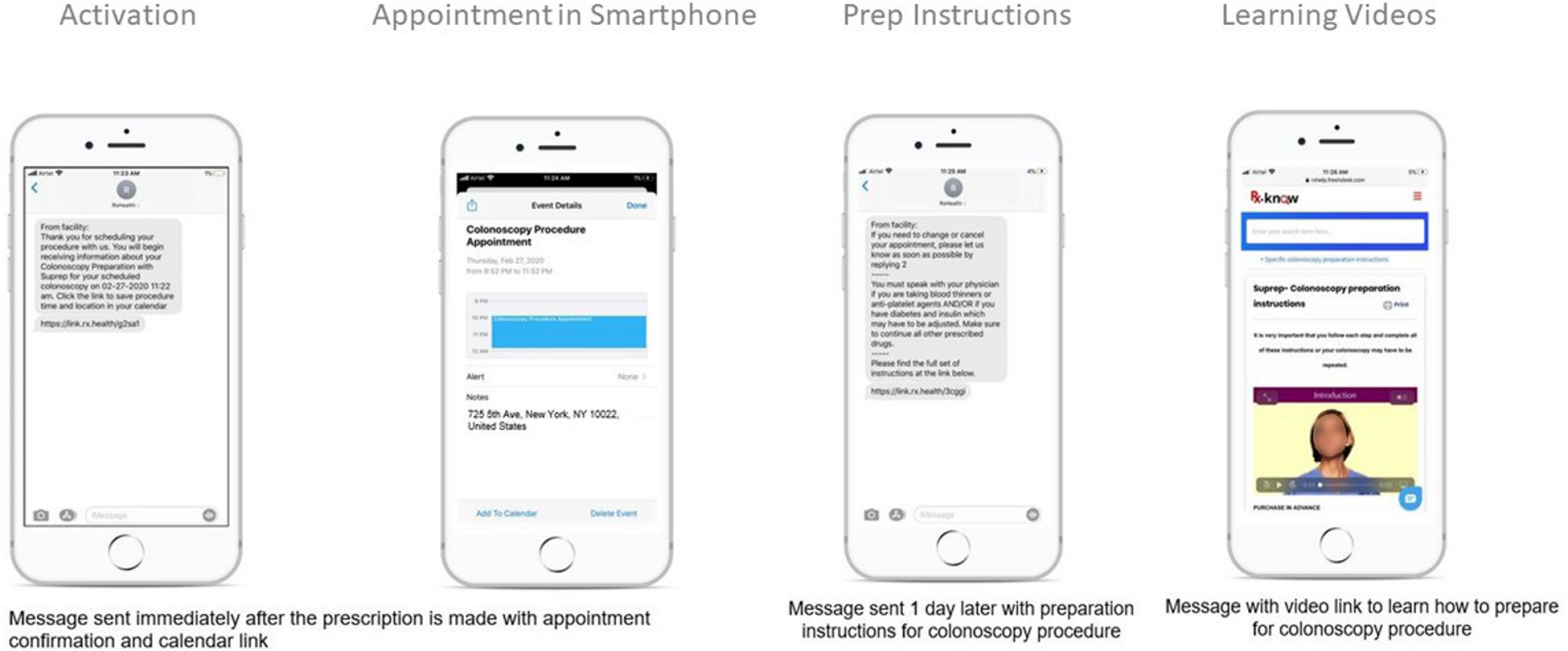
Patient view of PC-DNP instructions.

The PC-DNP group included patients who underwent a colonoscopy between February 2020 and May 2021. After scheduling a colonoscopy procedure date and time, patients/caregivers were asked by medical staff if they were interested in receiving the PC-DNP which would send them text-message reminders in preparation for their colonoscopy (Table 1 and Figure 2). In addition to undergoing a colonoscopy at The Valley Hospital and providing written consent for study participation, study inclusion criteria included proficiency of reading and speaking the English language, owning a smartphone, and willingness to receive the PC-DNP. The medical staff then initiated the enrollment in the DNP via the of their stool, and instructions on changing/canceling the appointment. The messages also included a link to the full set of bowel preparation instructions.

**Table 1:** PC-DNP texting schedule and message content for polyethylene glycol 3,350 and bisacodyl bowel preparation (20–30 kgs).

Schedule	Message Content
At the time of prescription	Thank you for scheduling your child's procedure with us. You will begin receiving information about Colonoscopy Preparation with polyethylene glycol 3,350 (Miralax®) and bisacodyl for your child's scheduled colonoscopy on <appointment date and time>.
	Click the link to save procedure time and location in your calendar.
15 min after prescription	To learn more about how to prepare your child for colonoscopy or your child had poor bowel preparation before, you should click here: <education module>. You can expect to be at the endoscopy center for 1 1/2–2 h.
10 days before at 9 am	If you need to change or cancel your child's colonoscopy appointment, please let us know as soon as possible by pressing 2.
	You must speak with your primary care physician or specialist if your child has diabetes and takes insulin which may have to be adjusted. Make sure to continue all other prescribed drugs.
	Please find the full set of instructions for your bowel prep at the link below: <link to instructions>
Triggered if #2 is pressed	Please call the number below to change your child's procedure appointment <phone number>
5 days before at 9 am	If you need to change or cancel your child's appointment, please let us know as soon as possible by pressing 2
Triggered if #2 is pressed	Please call the number below to change your child's procedure appointment <phone number>
4 days before at 9 am	Don’t offer your child bulk-forming agents like Metamucil®, iron supplements, and start him/her on a low fiber diet with no seeds, nuts, and dried fruits. Please purchase your child's preparation 2–4 days before the procedure. Do not mix the solution until the day before the procedure.
1 day before at 8 am	Your child's colonoscopy is tomorrow.
	Take one 238 gm of polyethylene glycol 3,350 bottle and mix 6 capfuls of polyethylene glycol 3,350 or one whole polyethylene glycol 3,350 OTC bottle in 24 ounces of liquid-like Pedialyte® or a sports drink that is blue or colorless Gatorade®, or Powerade® in a large pitcher. You may use any other clear electrolyte liquid you choose as well. Many people prefer to keep the mixture chilled. Please give your child 1 bisacodyl tablets or 1 suppository (5 mg) (you may crush the tablets if there is any difficulty in swallowing) at 12 pm the day before the procedure.
	Your child can have a light breakfast like bread and butter or milk. For the rest of today, only clear electrolyte liquid diet which includes: Apple or white grape juice, clear soup broth, tea (without milk or creamer), clear carbonated beverages such as ginger ale/lemon-lime soda, Gatorade® or other sports drinks, Kool-Aid® or other flavored drinks, coconut water, plain Jell-O® or other gelatins, popsicles. Avoid: red and purple colored foods
	Please find the full set of instructions once again in the link below:
1 day before at 12 pm	Please give your child 1 Dulcolax tablet or suppository (5 mg) (you may crush the tablets if you have any difficulty in swallowing).
1 day before at 2 pm	Your child may start drinking the polyethylene glycol 3,350 mixture every 10–15 min. You may adjust the time you begin drinking this dose to be later in the evening if you need to. You can expect it will take about 3–4 h for your child to drink this amount of fluid.
1 day before at 6 pm	Please give your child 1 Dulcolax tablet or suppository (5 mg) (you may crush the tablets if you have any difficulty in swallowing).
	Your child may have up to 5–10 watery bowel movements shortly thereafter, although some children may have little or no bowel movements. The output of stool at the end of the preparation should be watery, clear, greenish/yellowish with no solid or particulate matter. Your child may continue to drink clear electrolyte liquids until 8 h before you arrive for your procedure, but please do not let your child take anything by mouth after that.
1 day before at 9 pm	The stools should appear watery, clear, greenish/yellowish, your child may continue to drink clear electrolyte liquids until midnight. If still brown and pieces are seen please contact <phone number>
	If your prep is inadequate, the physician may not be able to visualize the entire colon adequately.
3 h before procedure	The stool output should be a yellow-tinged clear fluid at this time, without any particulate matter or solid stool. If you see solid stool or particulate matter, please press 2. You must arrive at least one hour before your scheduled appointment <appointment time and date>. Please find directions to the facility at the link below <facility address>
Triggered if #2 is pressed	Please call the number below to change your child's procedure appointment <phone number>
Night of procedure at 7 pm	Thank you for having your child's procedure with us today! If you have any concerns about how your child is feeling or any further questions about your procedure, please press 2
Triggered if #2 is pressed	Please call us at <phone number>
Next day of procedure at 9 am	Thank you again for having your child's procedure with us.
	To help us improve our service, please reply on a scale of 0-10, with 10 being “extremely likely”, how likely are you to recommend this digital program to a friend or family member who might need it?

The standard care group consisted of a historical sample of pediatric patients who underwent diagnostic and/or therapeutic colonoscopy between January 2018 and October 2020 at The Valley Hospital and received standard care instructions, given as written instructions, regarding bowel preparation and a phone call reminder of the upcoming procedure date and time. All comparison patients were selected using a random number generator and they were not contacted for this study. Patients were age and sex matched to the intervention group in order to minimize bias. Incomplete procedures were excluded from analysis.

### Data collection & analysis

Procedure indications and procedure outcomes were obtained through electronic health record (EHR) review. Similarly, bowel preparation quality (excellent/good, fair, or poor) as reported by the physician (proceduralist) was also obtained through EHR review. Those without a documented bowel preparation score were scored retrospectively based on images by one of the researchers. These outcomes were assessed using Pearson Chi-Square Tests, with *P* = .05 used as the threshold for statistical significance. This study was approved by the Institutional Review Board at The Valley Hospital.

## Results

### Population

One hundred seventeen pediatric patients (mean age = 13 y, range = 2–19) who underwent diagnostic and/or therapeutic colonoscopy at Valley Hospital met inclusion criteria for the study. The PC-DNP group consisted of 56 patients (mean age = 13 y, range = 3–19) and the standard care group consisted of 61 patients (mean age = 13 y, range = 2–18).

### Procedure indications and outcomes

There were no significant differences in procedure indications between the PC-DNP and standard care groups. Similarly, there were no significant differences in procedure outcomes, including polyp detection (Table 2).

**Table 2:** Procedure indications, outcomes and bowel preparation quality of standard care and PC-DNP groups.

Indication/Outcome	Standard Care (*n* = 61), *n*(%)	PC-DNP (*n* = 56), *n* (%)	*p* value
Abdominal Pain			0.68
Abdominal Pain	35 (57.4)	30 (53.6	
No Abdominal Pain	26 (42.6	26 (46.4)	
Diarrhea			0.53
Diarrhea	27 (44.3)	28 (50.0)	
No Diarrhea	34 (55.7)	28 (50.0)	
IBD			0.47
IBD	31 (50.8)	32 (57.1)	
No IBD	40 (49.2)	24 (42.9)	
Hematochezia			1.2
Hematochezia	14 (23.0)	18 (32.1)	
No Hematochezia	47 (77.0)	38 (67.9)	
Polyp			0.90
Polyp	2 (3.3)	4 (7.1)	
No Polyp	59 (96.7)	52 (92.9)	
Bowel Preparation Quality			0.0068*
Excellent/Good	51 (83.6)	55 (98.2)	
Fair	9 (14.8)	1 (1.8)	
Poor	1 (1.6)	9 (0)	

IBD, Inflammatory Bowel Disease.

* Statistically significant.

### Bowel preparation outcomes

**Figure 3:**
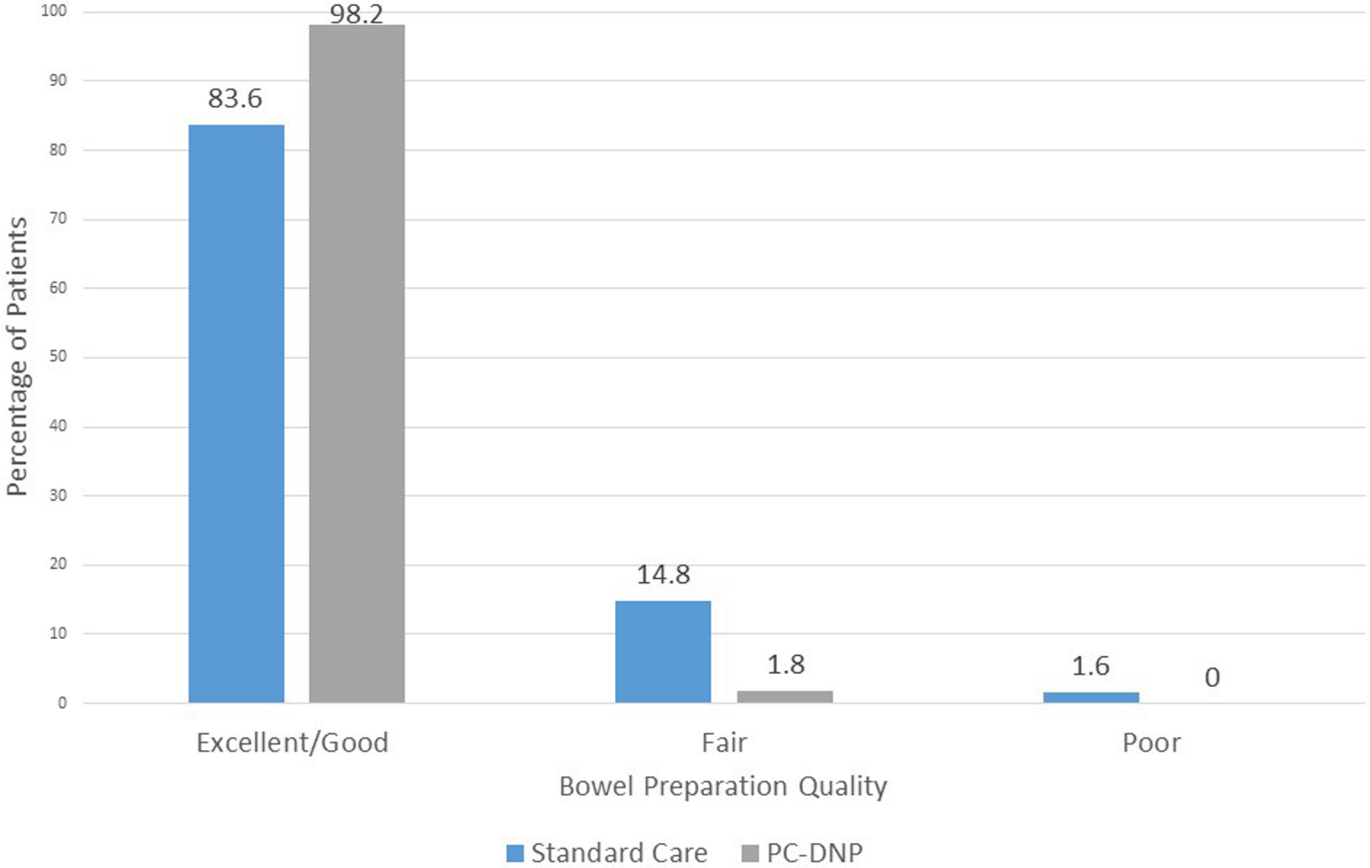
Bowel preparation quality comparison.

Of the 56 patients who were prescribed the PC-DNP, the bowel preparation success was recorded as excellent/good for 55 (98.2%) of the patients, and as fair for 1 (1.8%) patient (Table 2). Of the 61 patients who received standard care, the bowel preparation success was recorded as excellent/good for 51 (83.6%) patients, as fair for 9 (14.8%) patients, and as poor for 1 patient (1.6%). There was a significant difference in bowel preparation quality between the PC-DNP and the standard care groups (*P* < .01) (Table 2 and Figure 3).

## Discussion

The PC-DNP provided an automated channel for guiding pediatric patients through their colonoscopy preparation. We found that pediatric patients/caregivers who were prescribed the PC-DNP had significantly higher bowel preparation quality than patients who received standard care. These results demonstrate that automated DNPs such as the PC-DNP may be used to improve bowel preparation quality and completion rates for pediatric patients.

The PC-DNP addresses key barriers to adequate bowel preparation including difficulty understanding and/or misplacement of lengthy bowel preparation instructions. By sending patients/caregivers time-based text-messages with multi-media educational materials and specific instructions on bowel preparation, diet, and stool appearance, patients/caregivers were able to build a better understanding of proper bowel preparation, as evidenced by improved bowel preparation quality.

In this study, there were no significant differences in polyp detection between the PC-DNP and standard care groups, despite there being a significant difference in bowel preparation quality. This, however, may be attributed to the overall low incidence of polyp detection in pediatrics.

Multiple studies have demonstrated that improved bowel preparation leads to improved polyp detection and that in adults, DNPs similar to the PC-DNP can increase adenoma detection (6, 7). Additionally, the DNP in other studies has been shown to reduce the no-show rates in adults, reduce the rates of aborted procedures and increase patient satisfaction (4, 8). The PC-DNP are now expanded to support advanced endoscopy procedures as well as surgeries and imaging in many service lines.

Limitations of the study included lack of randomization between the two groups and our small sample size. This study was limited to a retrospective control group given the DNP had already been implemented successfully in our clinic. Additionally, the bowel preparation scoring was not documented by the proceduralist on all colonoscopy reports in both the DNP and control group, and was scored in these cases retrospectively and unblinded by one of the researchers which can introduce bias. The low incidence of polyp detection in pediatric colonoscopy in general limits polyp/adenoma detection rate as an outcome.

Another limitation of the study was the eligibility criteria of having a Smartphone, which may have limited its generalizability to those without digital skills or access to a Smartphone. Since the completion of study, the program has now expanded to include anyone with a cellular or landline. Patients who are not engaged or do not have a Smartphone can now get interactive voice response (IVR) to their phone along with an alert to practice staff to proactively follow up with the patient. Further studies are needed to assess impact of DNP in addressing digital health inequity and measuring overall savings by decreasing personnel costs through automation.

To our knowledge, this is one of few studies investigating the impact of DNPs on bowel preparation quality of pediatric patients (9). Our results provide evidence that DNPs such as the PC-DNP can be used to successfully improve pediatric patient colonoscopy preparation. Future studies are needed to further examine the benefits of DNPs on improving outcomes and satisfaction of pediatric patients across additional diseases and procedures.

## Data Availability

The raw data supporting the conclusions of this article will be made available by the authors, without undue reservation.
